# Supplementary material for: Development of a robust protocol for the characterization of the pulmonary microbiota
Source: Commun Biol. 2021 Feb 5;4:164. doi: 10.1038/s42003-021-01690-5 (PMC7864980; doi:10.1038/s42003-021-01690-5)
Supplement: Supplementary file 2 — Supplementary Information [file 42003_2021_1690_MOESM2_ESM.pdf]

Supplementary materials for the article:

Development of a Robust Protocol for the Characterization of the Pulmonary Microbiota

Nathan Dumont-Leblond[1,2], Marc Veillette[1], Christine Racine[1], Philippe Joubert[1,3], Caroline Duchaine[1,2*]

1. Centre de recherche de l'institut universitaire de cardiologie et de pneumologie de Québec, Quebec City (QC), Canada
2. Département de biochimie, de microbiologie et de bio-informatique, Faculté des sciences et de génie, Université Laval, Quebec City (QC), Canada
3. Département de Biologie moléculaire, biochimie médicale et pathologie, Université Laval, Quebec City (QC), Canada

*Corresponding author. E-mail : Caroline.Duchaine@bcm.ulaval.ca

This document includes:

Supplementary Figures 1 to 7

Supplementary Tables 1 to 3

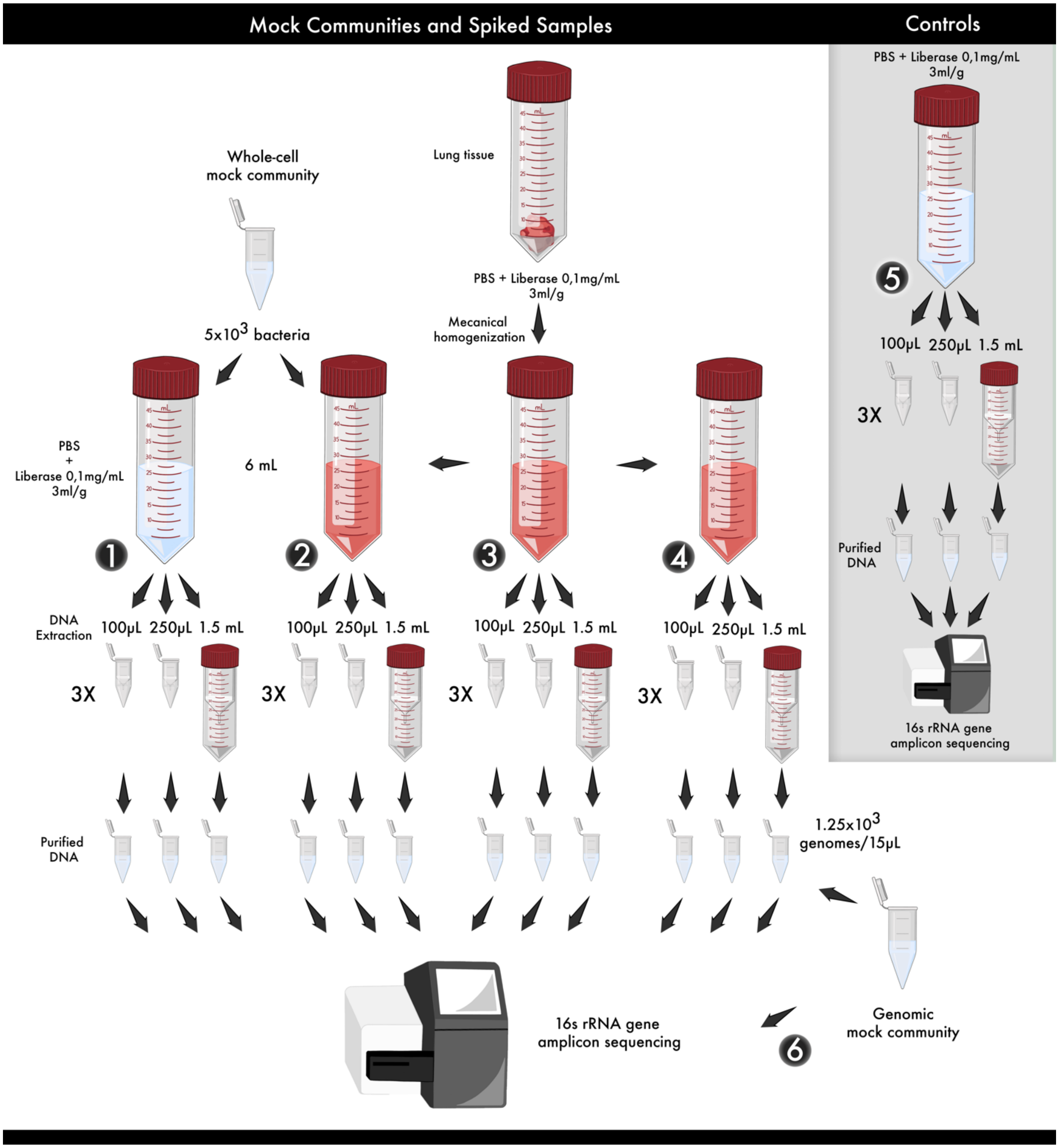

Supplementary Figure 1 : Detailed experimental design using microbial mock-communities.

Five types of samples or controls are shown. 1. Whole-cell community added to the enzymatic cocktail 2. Whole-cell community added to homogenized lung tissue, 3. Lung tissue extracted and sequenced by itself, 4. Homogenized lung tissue extract spiked with a genomic mock community, 5. Experimental control of enzymatic cocktail, 6. Genomic mock community sequenced by itself.

Supplementary Figure 2:
Microbial DNA extraction optimization pipeline tailored to the needs and constrains of pulmonary microbiota
Each steps of the experimental protocols are presented in detail from top to bottom, including who performs them and for approximately how long. Steps include patient selection, sampling and storage, homogenization, DNA extraction, sequencing and bioinformatics analysis. Refer to the methodology section of the article for more in-depth description. Blue shading represents storage periods. The total DNA of a same tissue homogenate or control is extracted using three different kits.

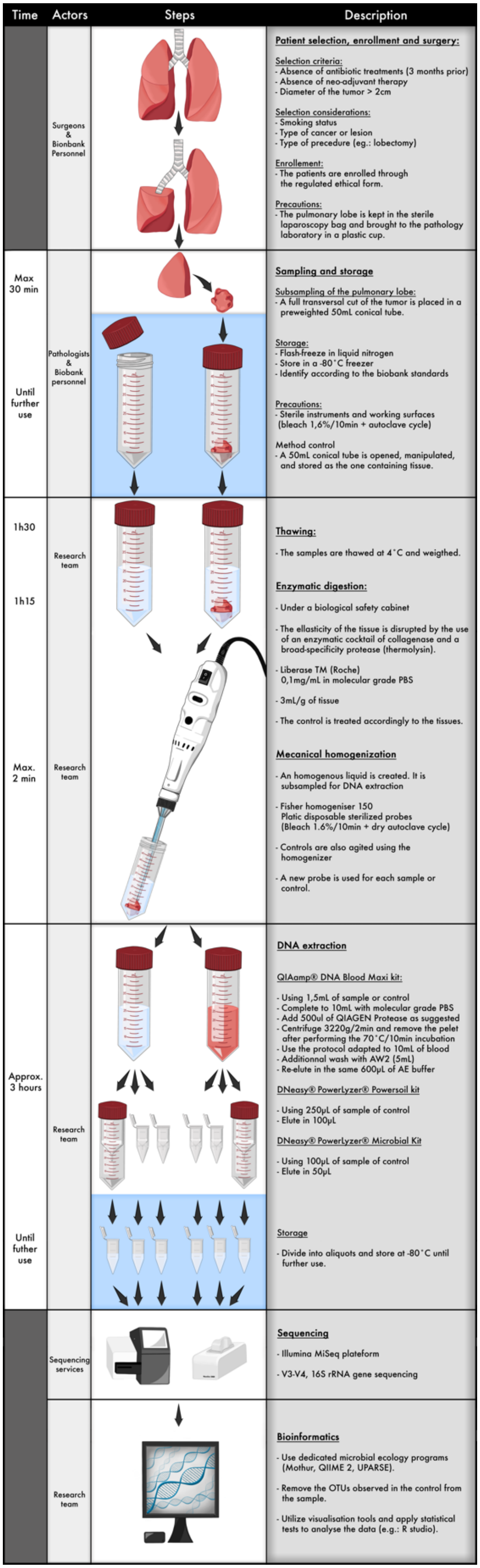

Supplementary Figure 3:
Final bacterial DNA extraction and sequencing pipeline tailored to the needs and constrains of pulmonary microbiota.
Each steps of the experimental protocols are presented in detail from top to bottom, including who performs them and for approximately how long. Steps include patient selection, sampling and storage, homogenization, DNA extraction, sequencing and bioinformatics analysis. Refer to the methodology section of the article for in-depth description. Blue shading represents storage periods.

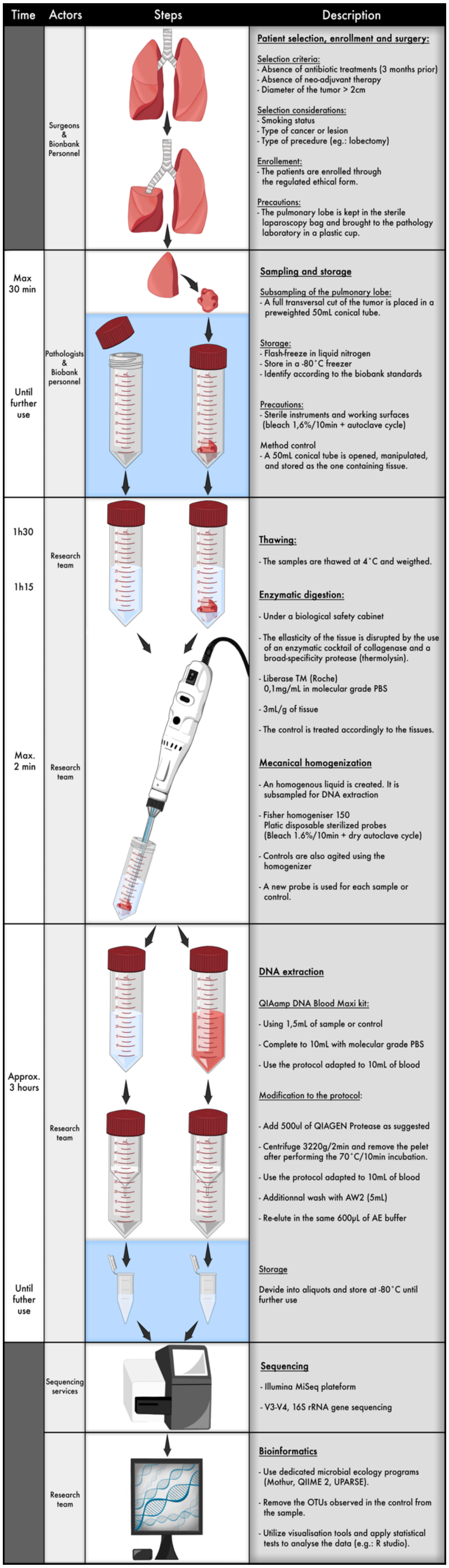

Supplementary Table 1. Patient's clinical data

Identification number (ID)	Age	Sexe	Smoking status	Lobe/Localization *	Tumor size, width/length (mm)	Histologic type*	Pathological stage
1	80	Female	Occasional smoker	RUL/Distal	31/22	A	1B
2	62	Female	Secondhand smoke	RLL/Distal	45/45	SCC	2A
3	77	Female	Never smoked	LLL/Distal	25/20	A	1A3
4	70	Male	Smoker (58 packs/year)	LLL/Distal	28/25	SCC	1B
5	78	Male	Smoker (54 packs/year)	RLL/Distal	70/70	SCC	3B

*LLL = left lower lobe, RLL = right lower lobe, RUL = right upper lobe, A = Adenocarcinoma, SCC = Squamous Cell Carcinoma

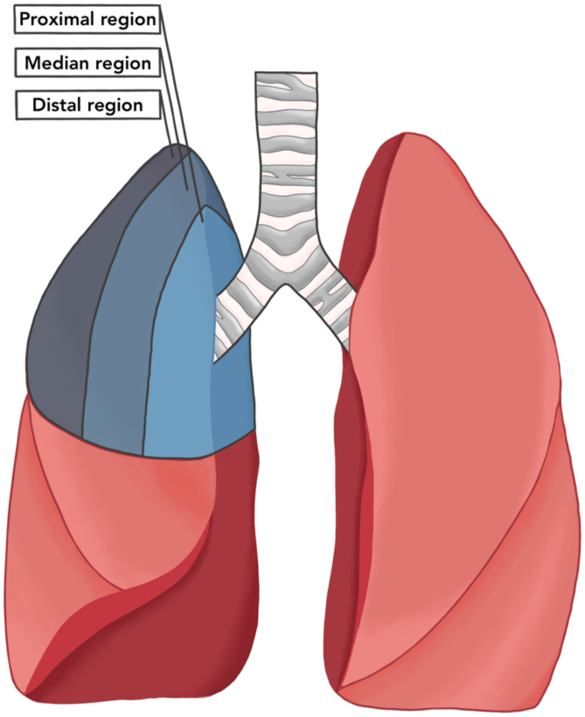

Supplementary Figure 4 : Pulmonary regions defined from the origin of the lobe.

Each lobe is divided in three sections based on their relative distance to the origin of the lobe bronchus, from the proximal to the distal region.

Supplementary Table 2: 16S rRNA gene V3-V4 primer sequences (1)

Primer ID	Sequence
Bakt_805R-long	CAAGCAGAAGACGGCATACGAGAT[index2]GTCTCGTGGGCTCGGAGAT GTGTATAAGAGACAGGACTACHVGGGTATCTAATCC
Bakt_341F-long	AATGATACGGCGACCACCGAGATCTACAC[index1]TCGTCGGCAGCGTC AGATGTGTATAAGAGACAGCCTACGGGNGGCWGCAG

Supplementary Table 3: Bacterial members of the mock communities (2,3)

Mock communities	Bacterial species
MSA-1002 & MSA-2002	*Acinetobacter baumannii*<br>*Actinomyces odontolyticus*<br>*Bacillus cereus*<br>*Bacteroides vulgatus*<br>*Bifidobacterium adolescentis*<br>*Clostridium beijerinckii*<br>*Cutibacterium acnes*<br>*Deinococcus radiodurans*<br>*Enterococcus faecalis*<br>*Escherichia coli*<br>*Helicobacter pylori*<br>*Lactobacillus gasseri*<br>*Neisseria meningitidis*<br>*Porphyromonas gingivalis*<br>*Pseudomonas aeruginosa*<br>*Rhodobacter sphaeroides*<br>*Staphylococcus aureus*<br>*Staphylococcus epidermidis*<br>*Streptococcus agalactiae*<br>*Streptococcus mutans*

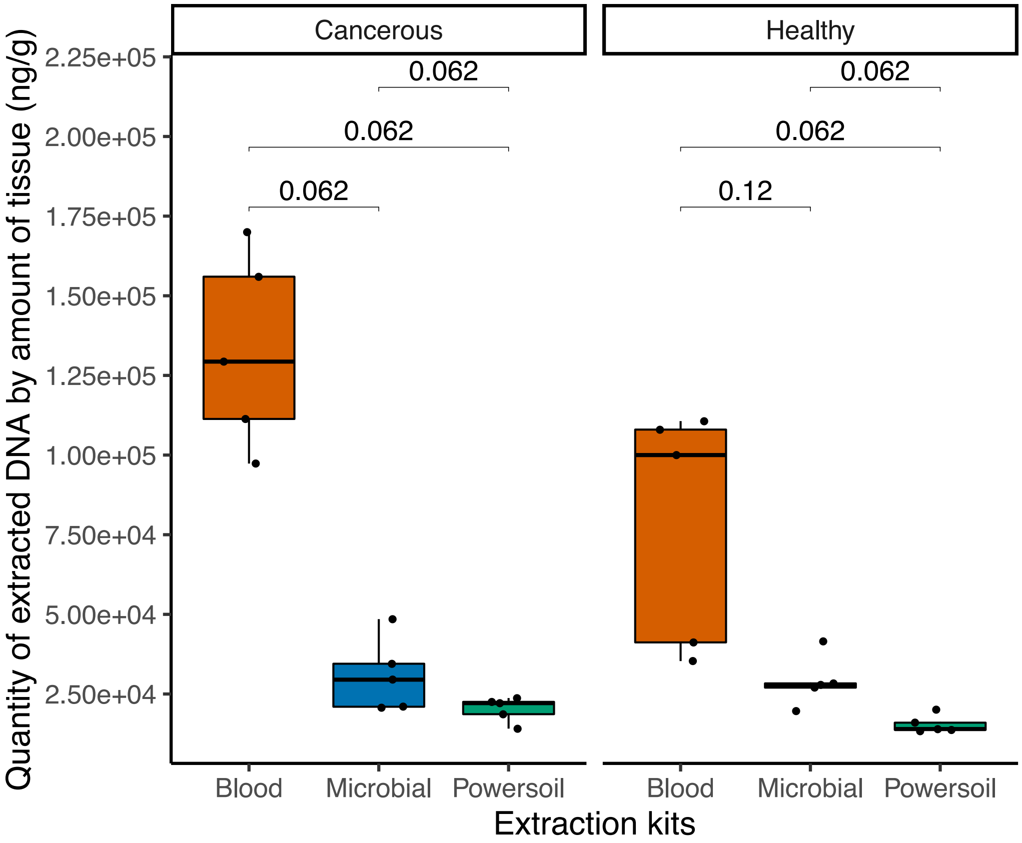

Supplementary Figure 5 :Quantity of extracted DNA by amount of tissue treated.

The concentration of DNA was detected by spectrometry at 260 nm for each type of tissue and extraction kits. The result was then multiplied by the volume of eluate and divided by the weight of tissue treated (0.333 g/mL). Double-sided paired samples Wilcoxon tests were performed to account for the lack of normal distribution and the patient variable. The boxes and bars display the data range, quartiles and median. n= 5 pairs of tissues (cancerous and healthy) from 5 different patients extracted by three distinct methods (total of 10 tissues).

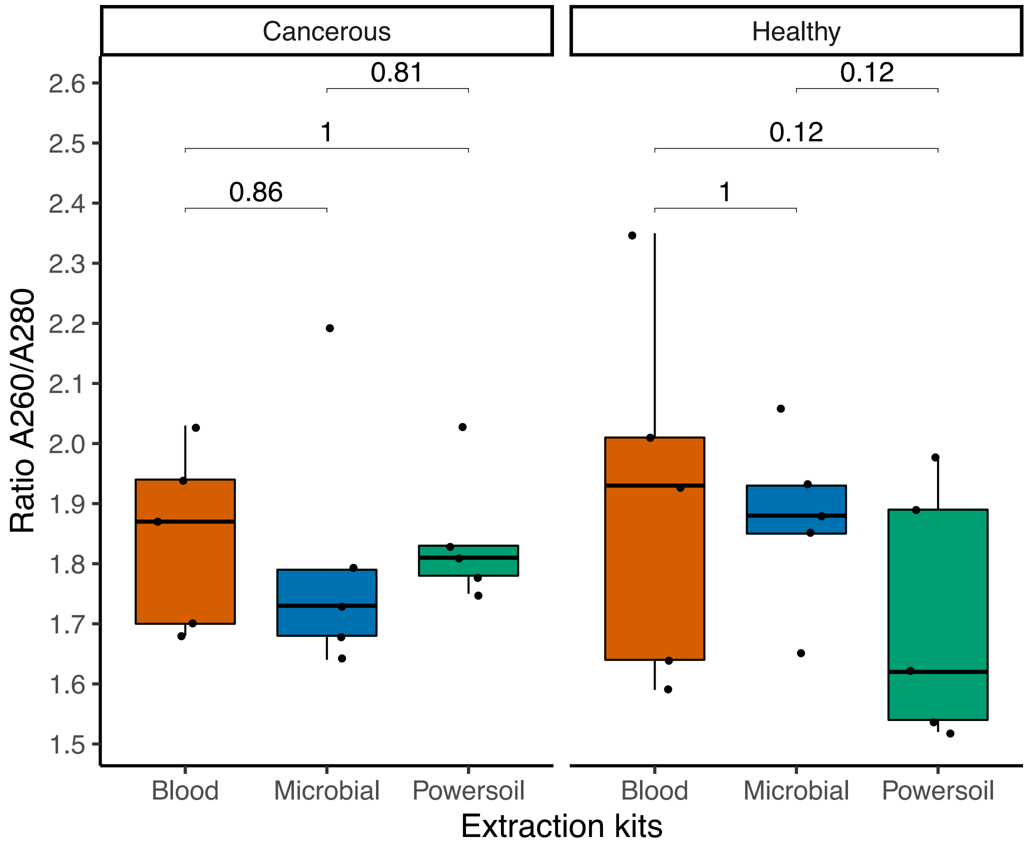

Supplementary Figure 6 :DNA purity of the eluates (280 nm).

The DNA purity was measured by spectrometry at 260 nm and 280 nm for each type of tissue and extraction kits. Double-sided paired samples Wilcoxon tests were performed to account for the lack of normal distribution and the patient variable. The boxes and bars display the data range, quartiles and median. n= 5 pairs of tissues (cancerous and healthy) from 5 different patients extracted by three distinct methods (total of 10 tissues).

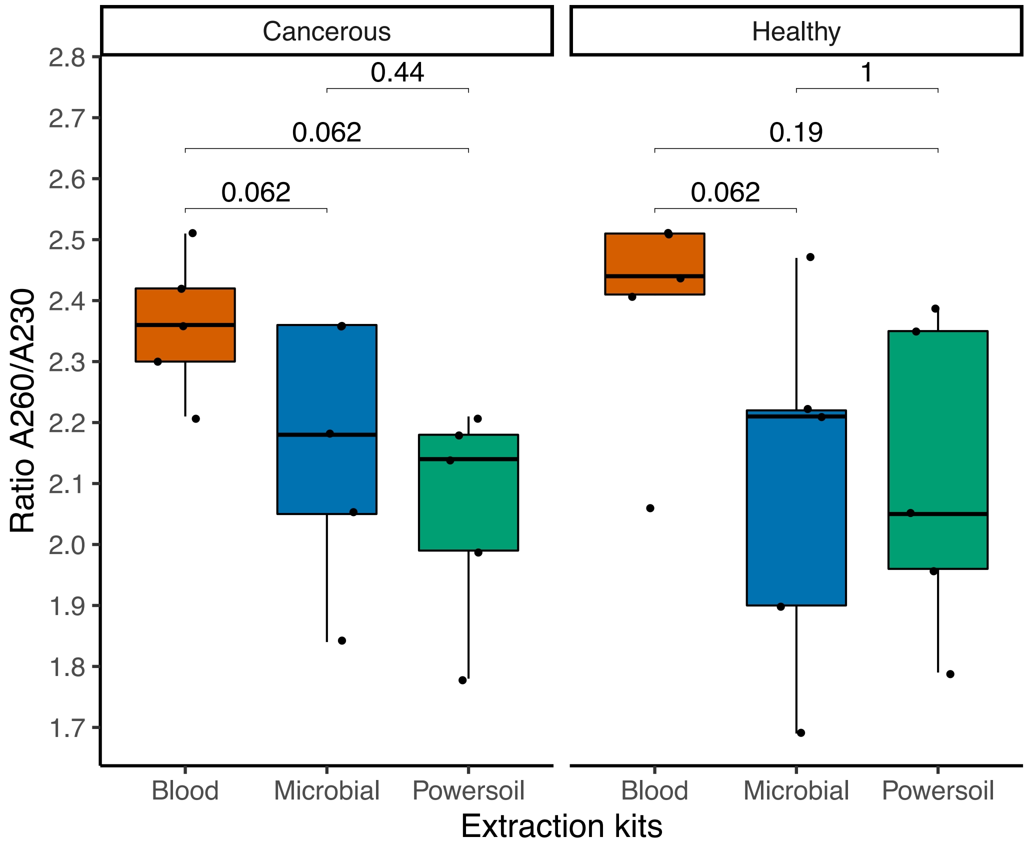

Supplementary Figure 7 : DNA purity of the eluates (230 nm).

The DNA purity was measured by spectrometry at 260 nm and 230 nm for each type of tissue and extraction kits. Paired samples Wilcoxon tests were performed to account for the lack of normal distribution and the patient variable. The boxes and bars display the data range, quartiles and median. n= 5 pairs of tissues (cancerous and healthy) from 5 different patients extracted by three distinct methods (total of 10 tissues).

Supplementary References:

1. Klindworth A, Pruesse E, Schweer T, Peplies J, Quast C, Horn M, et al. Evaluation of general 16S ribosomal RNA gene PCR primers for classical and next-generation sequencing-based diversity studies. Nucleic Acids Res [Internet]. 2013 Jan 1 [cited 2019 Sep 16];41(1):e1–e1. Available from: https://academic.oup.com/nar/article/41/1/e1/1164457
2. American Type Culture Collection (ATCC). ATCC ® MSA-1002™ [Internet]. [cited 2020 Oct 23]. Available from: https://www.atcc.org/products/all/MSA-1002.aspx
3. American Type Culture Collection (ATCC). ATCC ® MSA-2002™ [Internet]. [cited 2020 Oct 23]. Available from: https://www.atcc.org/products/all/MSA-2002.aspx
