# Supplementary material for: Development of a robust protocol for the characterization of the pulmonary microbiota
Source: Commun Biol. 2021 Feb 5;4:164. doi: 10.1038/s42003-021-01690-5 (PMC7864980; doi:10.1038/s42003-021-01690-5)
Supplement: Supplementary file 3 — Description of Additional Supplementary Files [file 42003_2021_1690_MOESM3_ESM.pdf]

Description of Additional Supplementary Files

File name: Supplementary Data 1

Description: Sequencing data identification of samples and experimental replicates available in the Bioproject PRJNA632856. The identification numbers refer to the type of samples in the Supplementary Figure 1.

File name: Supplementary Data 2

Description: Underlying data of the figures. This file holds the raw data used to create the figures and to perform the statistics found in this article. It includes alpha diversity metrics of tissue samples, DNA yield and purity measurements, and negative control-related data.
