# Supplementary material for: Development of a robust protocol for the characterization of the pulmonary microbiota
Source: Commun Biol. 2021 Feb 5;4:164. doi: 10.1038/s42003-021-01690-5 (PMC7864980; doi:10.1038/s42003-021-01690-5)
Supplement: Supplementary file 6 — Reporting summary [file 42003_2021_1690_MOESM6_ESM.pdf]

nature research

Corresponding author(s): Caroline Duchaine

Last updated by author(s): Dec 10, 2020

Reporting Summary

Nature Research wishes to improve the reproducibility of the work that we publish. This form provides structure for consistency and transparency in reporting. For further information on Nature Research policies, see our Editorial Policies and the Editorial Policy Checklist.

Statistics

For all statistical analyses, confirm that the following items are present in the figure legend, table legend, main text, or Methods section.

n/a	Confirmed	
☐	☒	The exact sample size (*n*) for each experimental group/condition, given as a discrete number and unit of measurement
☐	☒	A statement on whether measurements were taken from distinct samples or whether the same sample was measured repeatedly
☐	☒	The statistical test(s) used AND whether they are one- or two-sided<br>*Only common tests should be described solely by name; describe more complex techniques in the Methods section.*
☐	☒	A description of all covariates tested
☐	☒	A description of any assumptions or corrections, such as tests of normality and adjustment for multiple comparisons
☐	☒	A full description of the statistical parameters including central tendency (e.g. means) or other basic estimates (e.g. regression coefficient) AND variation (e.g. standard deviation) or associated estimates of uncertainty (e.g. confidence intervals)
☒	☐	For null hypothesis testing, the test statistic (e.g. *F*, *t*, *r*) with confidence intervals, effect sizes, degrees of freedom and *P* value noted<br>*Give P values as exact values whenever suitable.*
☒	☐	For Bayesian analysis, information on the choice of priors and Markov chain Monte Carlo settings
☒	☐	For hierarchical and complex designs, identification of the appropriate level for tests and full reporting of outcomes
☐	☒	Estimates of effect sizes (e.g. Cohen's *d*, Pearson's *r*), indicating how they were calculated

Our web collection on statistics for biologists contains articles on many of the points above.

Software and code

Policy information about availability of computer code

Data collection	No specialized data collection software were used.
Data analysis	Sequences cleaning, clustering in Operational taxonomic units (OTUs,) and taxonomical classifications were performed using Mothur's SOP version 1.40.5 (Kozich et al., 2013; Schloss and al., 2019; Schloss et al., 2009). Additional diversity analysis we conducted using RStudio (RStudio Inc., 2016). The 16S data was imported and manipulated using the phyloseq package, version 1.26.1 (McMurdie and Holmes, 2013). Plots were created using the packages ggplot2 version 3.2.0 and ggpubr version 3.2.0 and 0.2.1 respectively (Kassambara, 2019; Wickham, 2016). Alpha diversity analyses were performed with the vegan, version 2.5-5, and microbiome R packages, version 1.4.2 (Lahti et al., 2017; Oksanen et al., 2019). The Bray-Curtis distance and analyses of variance (ADONIS) were performed the Vegan package, version 2.5-5 (Oksanen et al., 2019). Venn diagrams were obtained using ampvis2 package, version 2.5.8 (Andersen et al., 2018).The Pearson correlation statistics were performed on the relative counts of samples and controls from the same patient before removal using the metagenomeSeq package version 1.24.1 (Paulson et al.). The normality of the data distribution was controlled using the Shapiro-Wilk test provided by the stats package, version 3.5.2 (R Core Team, 2018). Accordingly, paired Wilcoxon acknowledging for the patient and extraction technic, were performed with ggpubr, version 0.2.1 (Kassambara, 2019).

For manuscripts utilizing custom algorithms or software that are central to the research but not yet described in published literature, software must be made available to editors and reviewers. We strongly encourage code deposition in a community repository (e.g. GitHub). See the Nature Research guidelines for submitting code & software for further information.

Data

Policy information about availability of data

All manuscripts must include a data availability statement. This statement should provide the following information, where applicable:

- Accession codes, unique identifiers, or web links for publicly available datasets
- A list of figures that have associated raw data
- A description of any restrictions on data availability

The data is publicly available on the Sequence Read Archive (SRA) of the National Center for Biotechnology Information (NCBI). Accession number : PRJNA632856. The raw sequencing data, as well as, the DNA quantification and purity assessment data used to produce the plots and analyses are available.

Field-specific reporting

Please select the one below that is the best fit for your research. If you are not sure, read the appropriate sections before making your selection.

☒ Life sciences ☐ Behavioural & social sciences ☐ Ecological, evolutionary & environmental sciences

For a reference copy of the document with all sections, see nature.com/documents/nr-reporting-summary-flat.pdf

Life sciences study design

All studies must disclose on these points even when the disclosure is negative.

Sample size	Five patients were selected to validate the proposed method. The cancerous and healthy of the excised pulmonary lobe from each was samples. As no other study of this kind exist to our knowledge, we were enable to perform sample-size calculation based on empirical evidences. We decided on 5 patients to minimize the cost and use of biological material. This relatively small amount of patient still provided statistically significant result between the different method tested. An additional pair of cancerous and healthy tissues for two patients were combined to performed the tests using mock-communities.
Data exclusions	No data was excluded.
Replication	No attempt to replicate the exact optimization protocol was done. However, the method described here was used to analyse the bacterial content of pulmonary tissues in further research.
Randomization	Each version of the protocol was tested on the samples of every patient. No randomization was necessary.
Blinding	No distinct group were created as every sample received a combination of the same treatments.

Reporting for specific materials, systems and methods

We require information from authors about some types of materials, experimental systems and methods used in many studies. Here, indicate whether each material, system or method listed is relevant to your study. If you are not sure if a list item applies to your research, read the appropriate section before selecting a response.

Materials & experimental systems

n/a	Involved in the study	
☒	☐	Antibodies
☒	☐	Eukaryotic cell lines
☒	☐	Palaeontology and archaeology
☒	☐	Animals and other organisms
☐	☒	Human research participants
☒	☐	Clinical data
☒	☐	Dual use research of concern

Methods

n/a	Involved in the study	
☒	☐	ChIP-seq
☒	☐	Flow cytometry
☒	☐	MRI-based neuroimaging

Human research participants

Policy information about studies involving human research participants

Population characteristics	Patients undergoing lung resection (lobectomy) for pulmonary cancer were selected (adenocarcinoma or squamous cell carcinoma). They were between the ages of 62 and 80 years old. Three patients are female, two male. They did not follow any antibiotic treatment in the three months prior to the surgery, nor did they receive a neoadjuvant therapy. Only patient with tumor of diameter superior to 2.0cm were enrolled.

Recruitment	Patient were recruited based on the inclusion criteria cited above through the Institut Universitaire de Cardiologie et de Pneumologie de Québec (IUCPQ) Biobank between September 2018 and November 2019.
Ethics oversight	Institut Universitaire de Cardiologie et de Pneumologie de Québec (IUCPQ) ethic committee (project #1200)

Note that full information on the approval of the study protocol must also be provided in the manuscript.
